# Supplementary material for: Targeting Fear of Cancer Recurrence with Internet-Based Emotional Freedom Techniques (iEFT) and Mindfulness Meditation Intervention (iMMI) (BGOG-gyn1b/REMOTE)
Source: Brain Sci. 2025 Aug 22;15(9):900. doi: 10.3390/brainsci15090900 (PMC12467542; doi:10.3390/brainsci15090900)
Supplement: Supplementary file 1 [file brainsci-15-00900-s001.zip › brainsci-3767384-supplementary.pdf]

Study Protocol

Targeting Fear of Cancer Recurrence with internet-based Emotional Freedom Techniques (iEFT) and Mindfulness Meditation Intervention (iMMI) (BGOG-gyn1b/REMOTE)

Supplementary Materials

SA. Table S1. Schematic overview of the data collection and interventions

.

	Visits				
**Procedures**	**Screening**	**T0***	**T1***	**T2***	**T3***
**Healthcare professional at local site**					
Informed consent	X				
Inclusion/exclusion criteria check	X				
Completion eCRF	X				
Randomisation		X			
**Central data manager**					
Adverse event check		X	X	X	X
Enrolment visit		X			
**Participant**					
Collection of hair sample[1]		X	X		
Performance of intervention strategy[2]		⟷		X[3]	X[3]
Completion of evaluation questionnaires		X	X	X	X

Abbreviations: eCRF: electronic Case Report Form.

* T0: baseline (i.e. before randomisation); T1: 6 weeks after start of the iEFT or iMMI programme or 6 weeks after randomisation to the wait-list; T2: 6 weeks after end of the iEFT or iMMI programme or 12 weeks after randomisation to the wait-list; T3: 24 weeks after start of the iEFT or iMMI programme or 24 weeks after randomisation to the wait-list.

[1] Optional, needs to be indicated in the Informed Consent Form (ICF).

[2] When assigned to the iEFT or iMMI group.

[3] Optional, participants may continue the intervention strategy after T1.

SB. Table S2: Participating hospitals

Hospital	Principal Investigator
az groeninge (coordinating centre; Kortrijk, Belgium)	Prof. Philip Debruyne
Antwerp University Hospital (Antwerp, Belgium)	Dr. Laure-Anne Teuwen
AZ Glorieux (Ronse, Belgium)	Dr. Florence Van Ryckeghem
AZ Klina (Brasschaat, Belgium)	Dr. Christine Langenaeken
AZ Vesalius (Tongeren, Belgium)	Dr. Katherine Vandenborre
Brussels University Hospital (Brussels, Belgium)	Dr. Christel Fontaine
Ghent University Hospital (Ghent, Belgium)	Prof. Tom Boterberg
Imelda ziekenhuis (Bonheiden, Belgium)	Dr. Heidi Van den Bulck
Jan Yperman Ziekenhuis (Ypres, Belgium)	Dr. Kurt Geldhof
Jessa ziekenhuis (Hasselt, Belgium)	Prof. Jeroen Mebis
Leuven University Hospital (Leuven, Belgium)	Prof. Thaïs Baert
VITAZ (Sint-Niklaas, Belgium)	Dr. Caroline Lamot

SC. Table S3: Data to be collected at baseline in the electronic case report form (eCRF)

**Socio-demographic data**
Date of birth, age and age category
Gender

Social status
Number of sons/daughters
Living situation
Highest education and age of graduation
Profession
**Medical data**
Date of diagnosis
Type of malignancy with specification
Histological type
Differentiation
TNM classification
Stage at diagnosis
Type of treatment with start date, end date (if applicable), and number of sessions (if applicable)
ECOG performance status
age-adjusted Charlson Comorbidity Index

Section SD. Patient Reported Outcomes Measures (PROMs)

- Fear of Cancer Recurrence Inventory (FCRI) [33, 34] (Appendix B): Simard and Savard developed the FCRI, a 42-item self-report instrument inspired by a cognitive-behavioural conceptualisation of FCR and intended for use with adults affected by cancer. The FCRI was developed to provide a standardised method of assessing individuals' fears about cancer recurrence and their ability to manage these fears along seven domains: triggers, severity, psychological distress, functional impairment, reassurance, insight and coping strategies [33]. The total score of the 42-item scale is typically interpreted as an overall measure of FCR, with a possible range from 0 to 168, with higher scores indicative of greater FCR [33]. Triggers (8 items) assesses the presence of stimuli that can activate FCR. Severity (9 items) evaluates the presence and severity of thoughts or images associated with FCR. Psychological Distress (4 items) and Functional Impairment (6 items) measure potential consequences of FCR. Insight (3 items) assesses the level of self-criticism towards FCR intensity. Reassurance (3 items) and Coping Strategies (9 items) measure coping responses that may influence FCR severity (e.g. denial, wishful thinking). Items are rated on a 5-point scale, where 0 = never/not at all, 1=rarely/a little, 2 =sometimes/somewhat, 3=most of the time/a lot and 4 = all the time/a great deal [33]. A score of ≥13 on severity (possible range: 0–36) is considered indicative of a fear response warranting clinical assessment [34]. High internal consistency (α = 0.95) and temporal stability (r = 0.89) have been demonstrated [34].
- Distress thermometer (DT) and 38-item Problem List [35, 36]: The DT has been widely used in psycho-oncology research internationally and is recommended as a routine clinical screening tool in oncology settings to detect clinically significant distress, indicated by a score of 4 or more [35]. To enhance its utility, a list of common concerns, called the "Problem List" (PL), was added to the DT to identify potential sources of distress [36]. The PL allows personalized triage by facilitating referral to the most appropriate support services [36].
- EORTC Quality of Life Survivorship Questionnaire Cancer Survivorship Core questionnaire: QLQ-SURV100 [37, 38], amended with items from the EORTC Core Quality of Life questionnaire (EORTC QLQ-C30) [39, 40]: in this RCT, we aim to focus on the health-related quality of life (HRQOL) of cancer survivors and will therefore apply the latest developed questionnaire, i.e. the SURV100 developed by the EORTC. This questionnaire captures the full range of physical, mental, and social HRQOL issues relevant to disease-free cancer survivors [37]. Items are rated on a 4-point scale, where 0 = never/not at all, 1=sometimes/somewhat, 3=most of the time/a lot and 4 = all the time/a great deal. Currently, a validation study is ongoing in different countries [37]. By the end of this trial, we aim to have a score indicative for HRQOL. To be able to completely cover

all health-related QoL aspects, few items of the EORTC QLQ-C30 were amended to this scale [40]. Also, we aim to calculate the summary score as this may serve as a prognostic factor for survival of patients with cancer [41].

- EuroQol EQ-5D-5L [42]: Health status and quality-adjusted life year can be measured by using the outcomes of the EQ-5D-5L questionnaire. The descriptive system comprises five dimensions: mobility, self-care, usual activities, pain/discomfort and anxiety/depression [42]. Each dimension has 5 levels: no problems, slight problems, moderate problems, severe problems and extreme problems [42]. Participants are asked to indicate their health status by selecting the box corresponding to the statement that best describes their condition, for each of the five dimensions. Each response generates a single-digit score which can be combined into a 5-digit number that describes the patient's health status. In addition to the EQ-5D descriptive system, the questionnaire also incorporates the EQ visual analogue scale (EQ VAS) [42]. The EQ VAS records the patient's self-assessed health using a vertical visual analogue scale, with endpoints labelled 'The best health you can imagine' and 'The worst health you can imagine'. This scale can be used as a quantitative measure of health outcome based on the individual's own perception and judgement [42].
